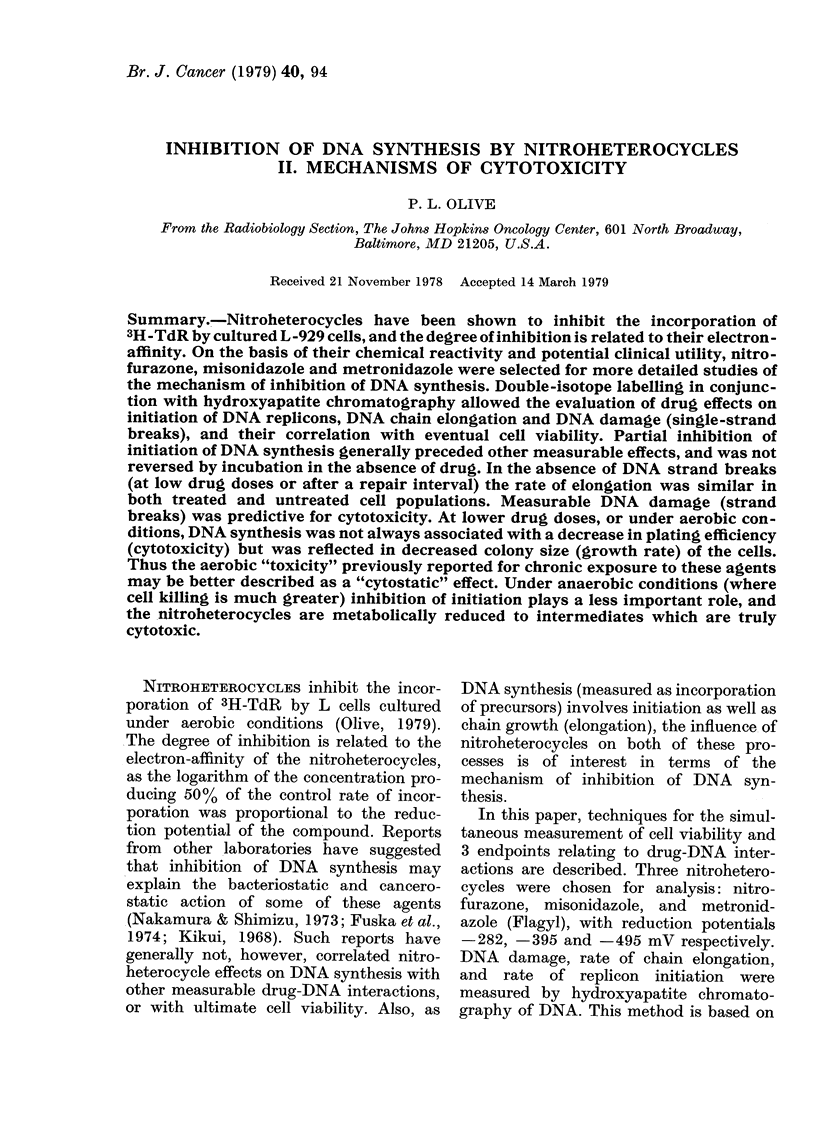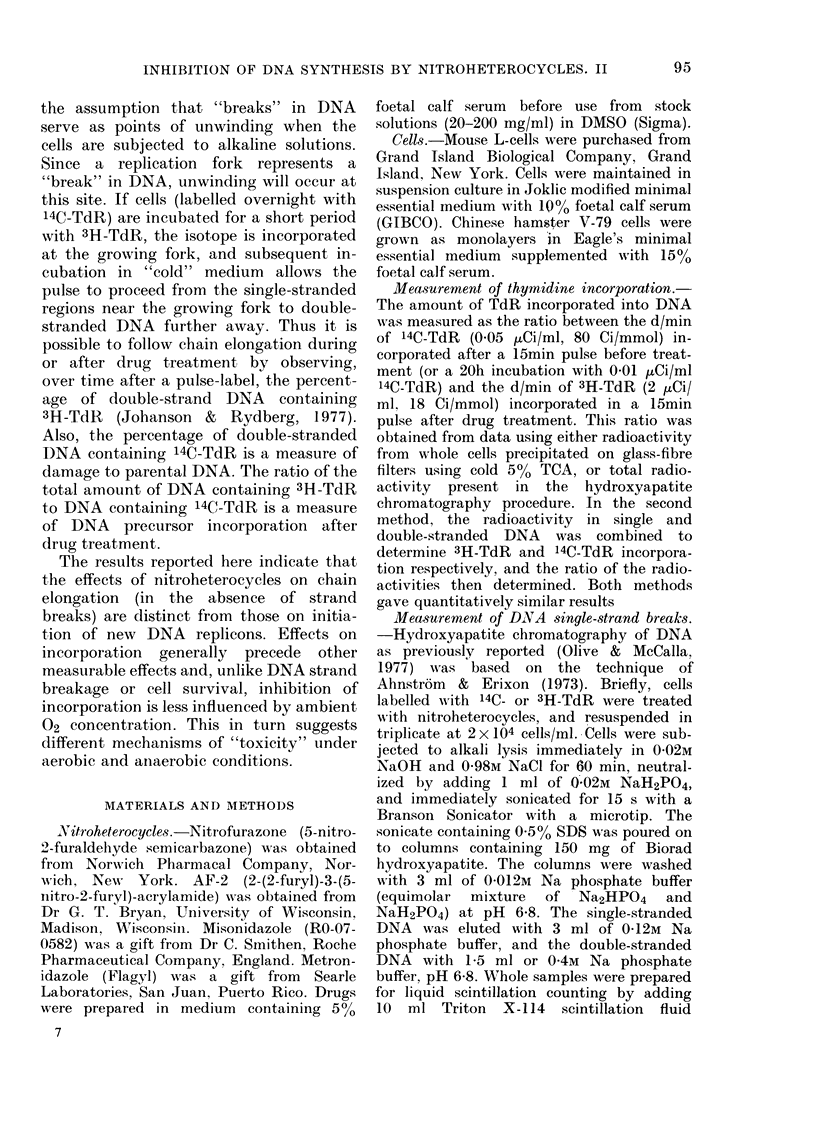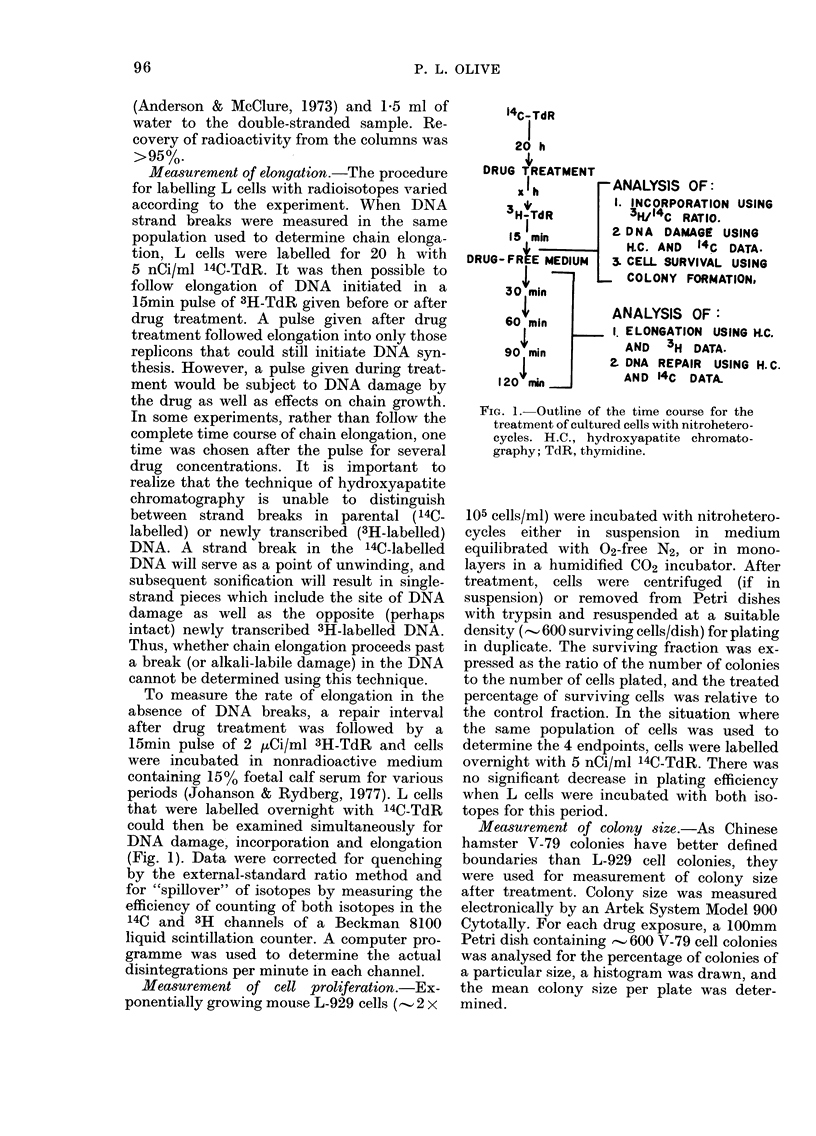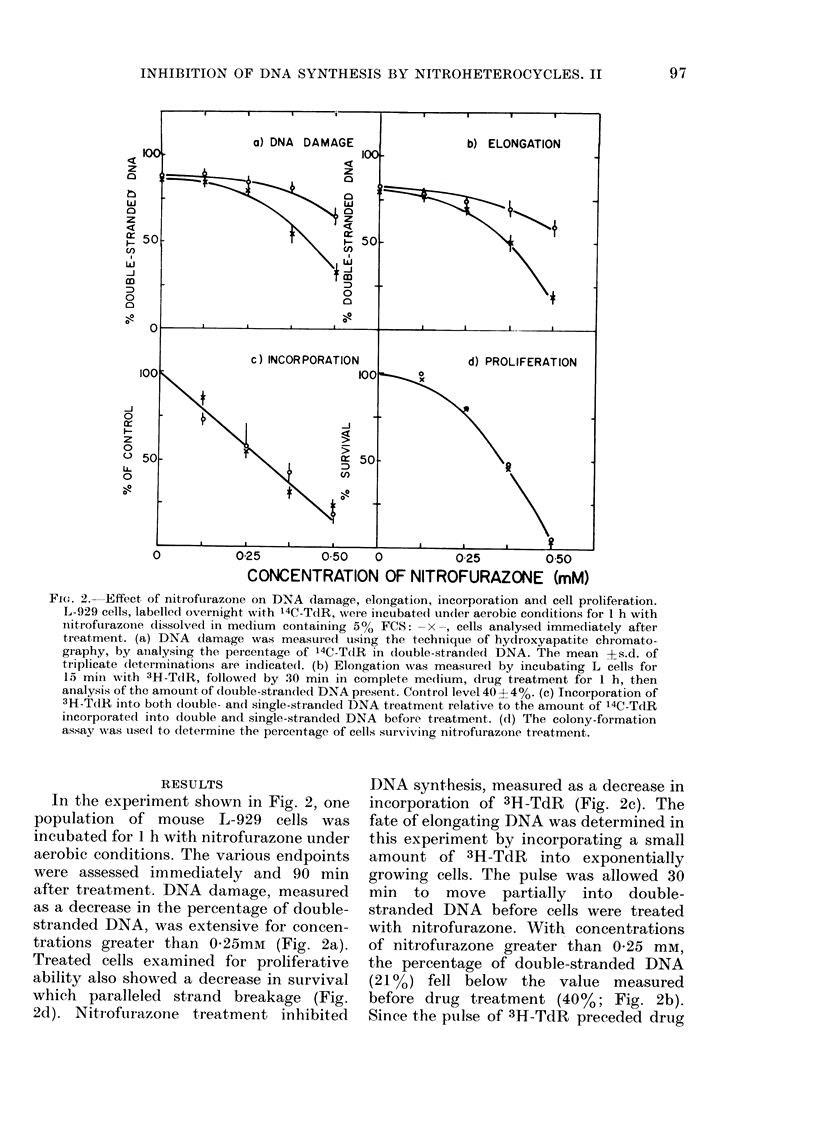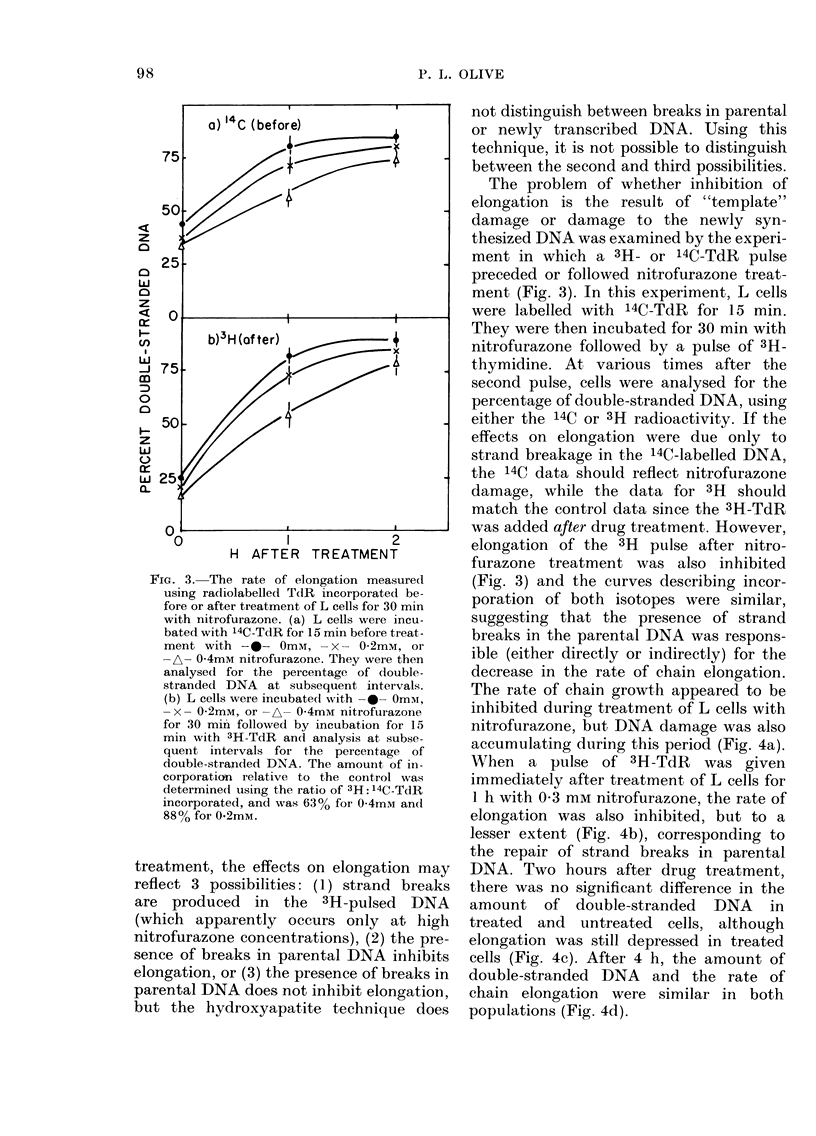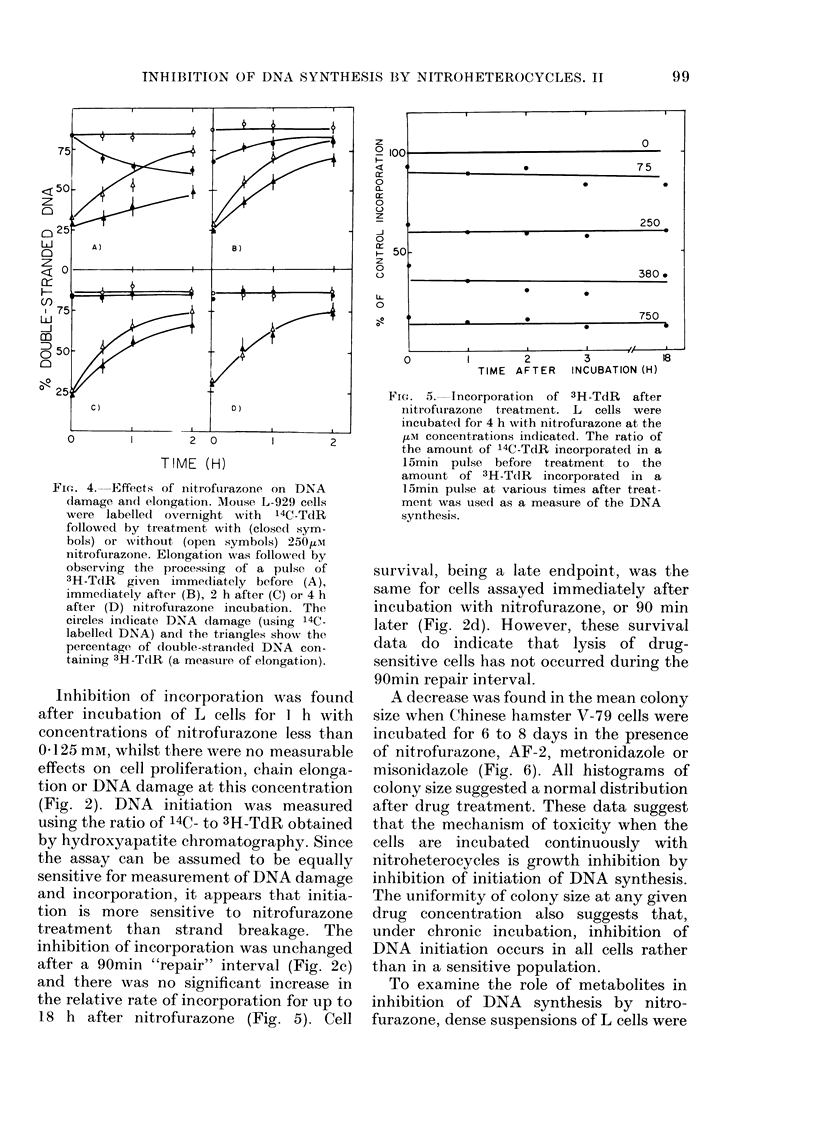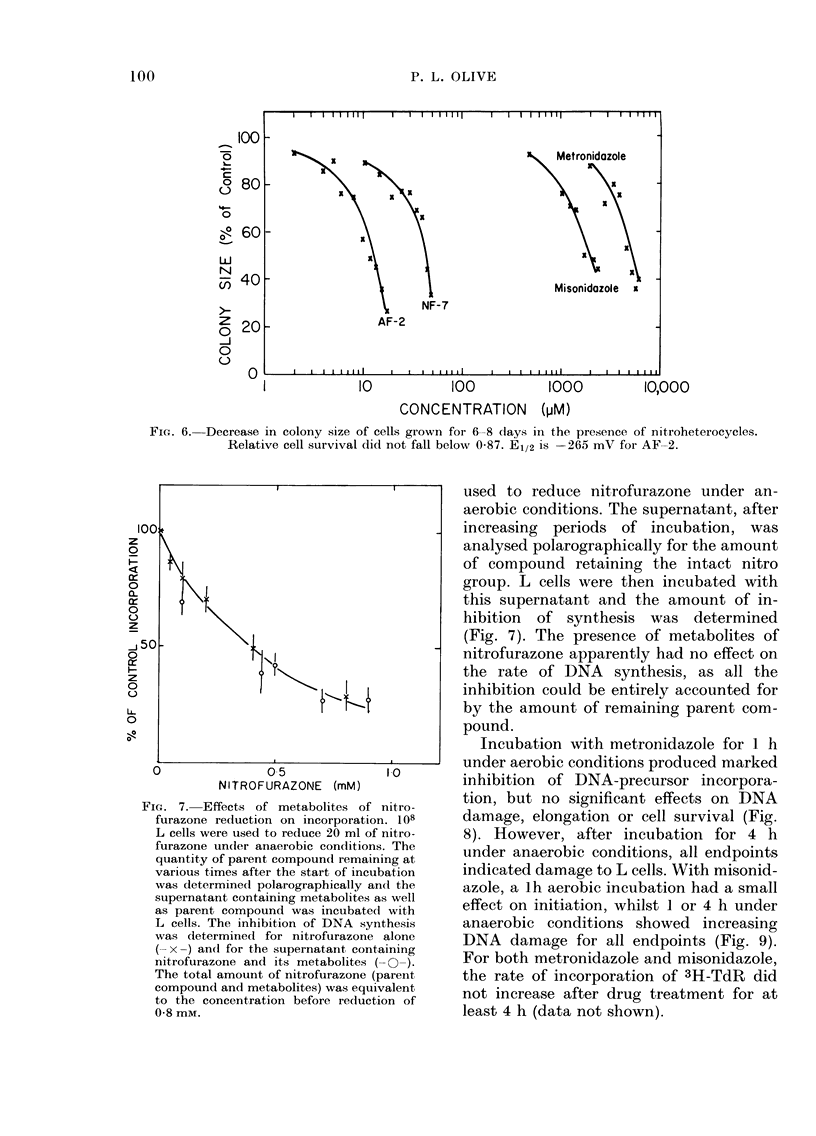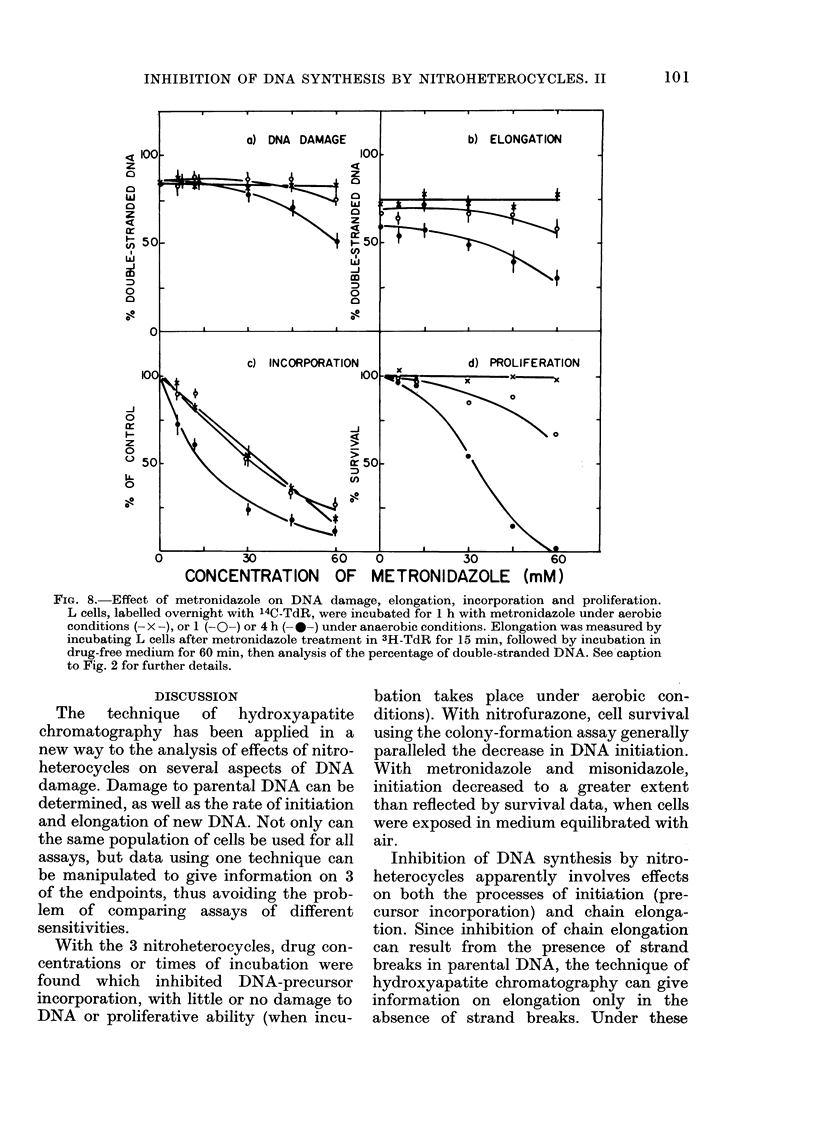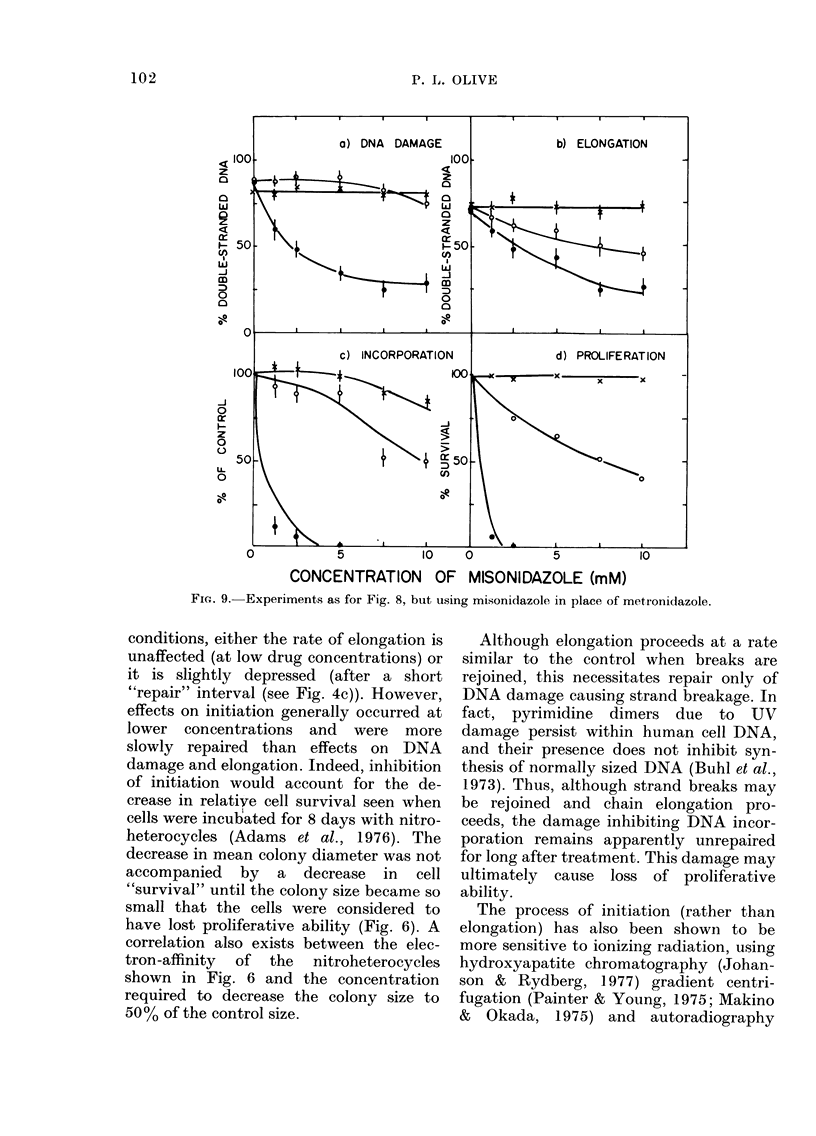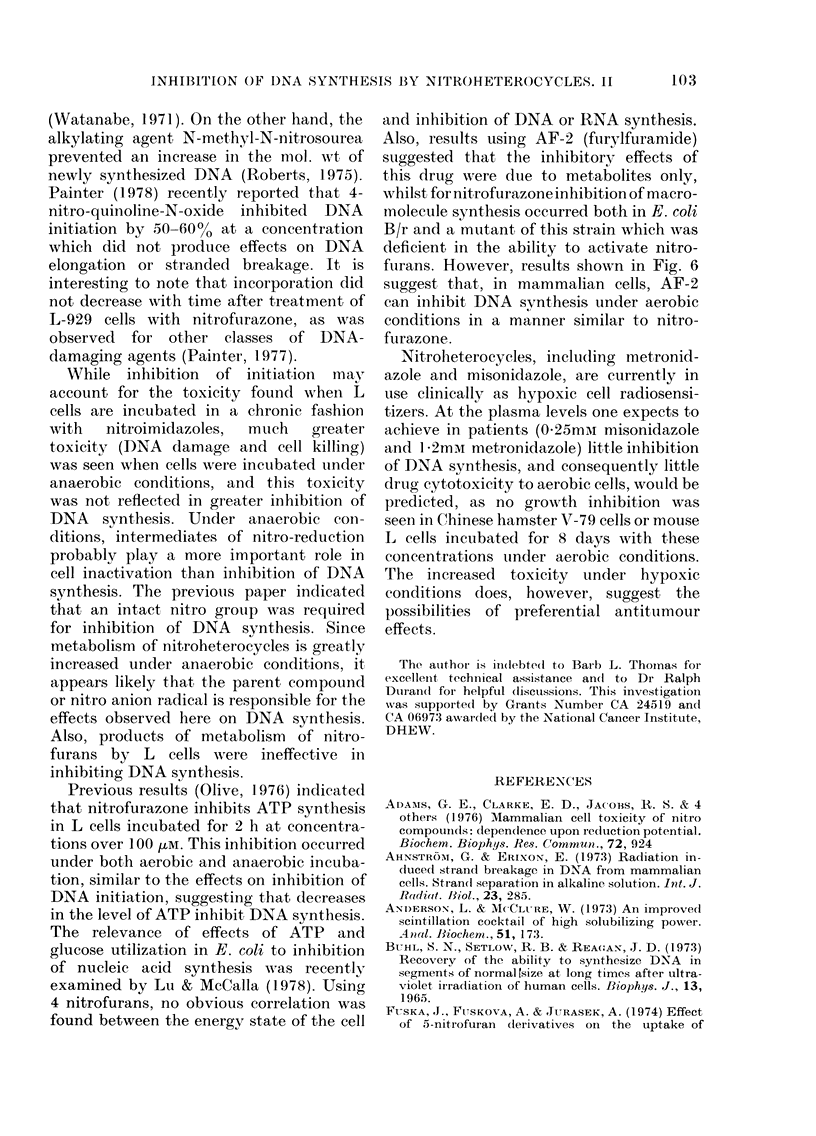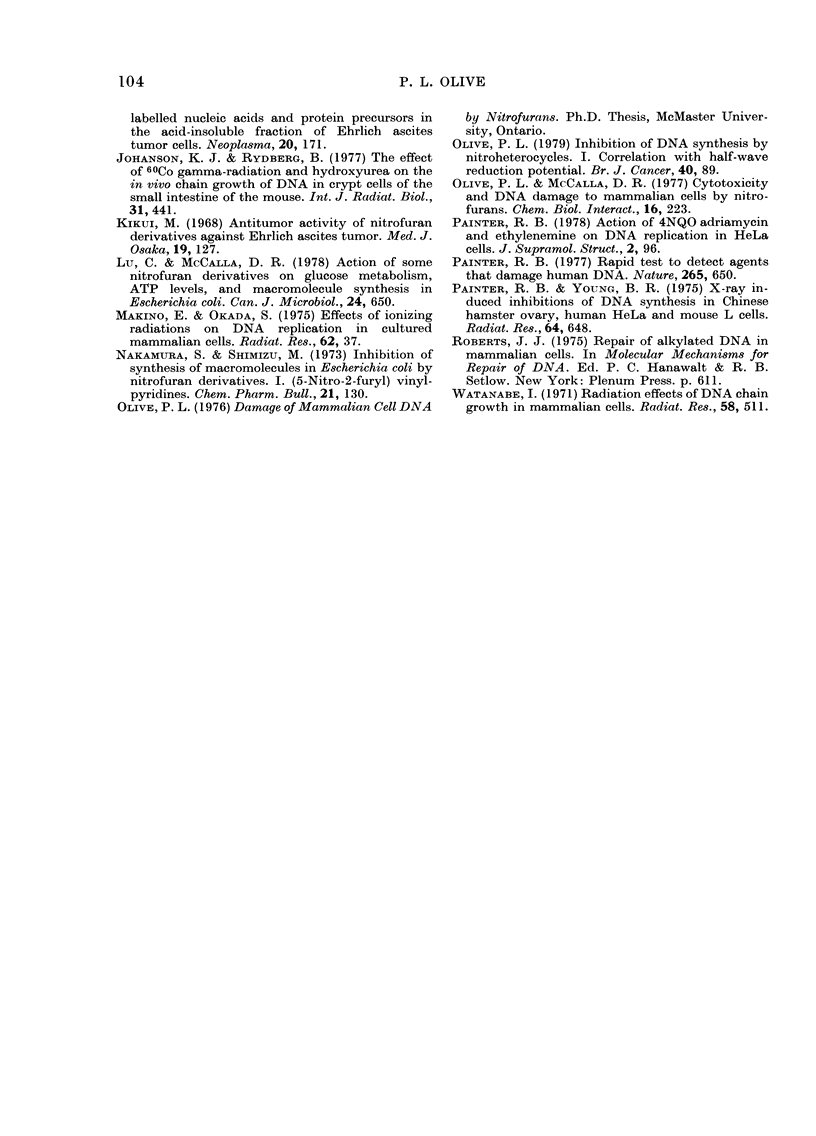# Inhibition of DNA synthesis by nitroheterocycles. II. Mechanisms of cytotoxicity.

**DOI:** 10.1038/bjc.1979.145

**Published:** 1979-07

**Authors:** P. L. Olive

## Abstract

Nitroheterocycles have been shown to inhibit the incorporation of 3H-TdR by cultured L-929 cells, and the degree of inhibition is related to their electron-affinity. On the basis of their chemical reactivity and potential clinical utility, nitrofurazone, misonidazole and metronidazole were selected for more detailed studies of the mechanism of inhibition of DNA synthesis. Double-isotope labelling in conjunction with hydroxyapatite chromatography allowed the evaluation of drug effects on initiation of DNA replicons, DNA chain elongation and DNA damage (single-strand breaks), and their correlation with eventual cell viability. Partial inhibition of initiation of DNA synthesis generally preceded other measurable effects, and was not reversed by incubation in the absence of drug. In the absence of DNA strand breaks (at low drug doses or after a repair interval) the rate of elongation was similar in both treated and untreated cell populations. Measurable DNA damage (strand breaks) was predictive for cytotoxicity. At lower drug doses, or under aerobic conditions, DNA synthesis was not always associated with a decrease in plating efficiency (cytotoxicity) but was reflected in decreased colony size (growth rate) of the cells. Thus the aerobic "toxicity" previously reported for chronic exposure to these agents may be better described as a "cytostatic" effect. Under anaerobic conditions (where cell killing is much greater) inhibition of initiation plays a less important role, and the nitroheterocycles are metabolically reduced to intermediates which are truly cytotoxic.


Br. J. Cancer (1979) 40, 94

### INHIBITION OF DNA SYNTHESIS BY NITROHETEROCYCLES II. MECHANISMS OF CYTOTOXICITY

P. L. OLIVE

*From the Radiobiology Section, The Johns Hopkins Oncology Center,* 601 *North Broadway, Baltimore, MD* 21205, *U.S.A.*

Received 21 November 1978 Accepted 14 March 1979

**Summary.—Nitroheterocycles have been shown to inhibit the incorporation of $^3$H-TdR by cultured L-929 cells, and the degree of inhibition is related to their electron-affinity. On the basis of their chemical reactivity and potential clinical utility, nitrofurazone, misonidazole and metronidazole were selected for more detailed studies of the mechanism of inhibition of DNA synthesis. Double-isotope labelling in conjunction with hydroxyapatite chromatography allowed the evaluation of drug effects on initiation of DNA replicons, DNA chain elongation and DNA damage (single-strand breaks), and their correlation with eventual cell viability. Partial inhibition of initiation of DNA synthesis generally preceded other measurable effects, and was not reversed by incubation in the absence of drug. In the absence of DNA strand breaks (at low drug doses or after a repair interval) the rate of elongation was similar in both treated and untreated cell populations. Measurable DNA damage (strand breaks) was predictive for cytotoxicity. At lower drug doses, or under aerobic conditions, DNA synthesis was not always associated with a decrease in plating efficiency (cytotoxicity) but was reflected in decreased colony size (growth rate) of the cells. Thus the aerobic "toxicity" previously reported for chronic exposure to these agents may be better described as a "cytostatic" effect. Under anaerobic conditions (where cell killing is much greater) inhibition of initiation plays a less important role, and the nitroheterocycles are metabolically reduced to intermediates which are truly cytotoxic.**

NITROHETEROCYCLES inhibit the incorporation of $^3$H-TdR by L cells cultured under aerobic conditions (Olive, 1979). The degree of inhibition is related to the electron-affinity of the nitroheterocycles, as the logarithm of the concentration producing 50% of the control rate of incorporation was proportional to the reduction potential of the compound. Reports from other laboratories have suggested that inhibition of DNA synthesis may explain the bacteriostatic and cancerostatic action of some of these agents (Nakamura & Shimizu, 1973; Fuska *et al.*, 1974; Kikui, 1968). Such reports have generally not, however, correlated nitroheterocycle effects on DNA synthesis with other measurable drug-DNA interactions, or with ultimate cell viability. Also, as DNA synthesis (measured as incorporation of precursors) involves initiation as well as chain growth (elongation), the influence of nitroheterocycles on both of these processes is of interest in terms of the mechanism of inhibition of DNA synthesis.

In this paper, techniques for the simultaneous measurement of cell viability and 3 endpoints relating to drug-DNA interactions are described. Three nitroheterocycles were chosen for analysis: nitrofurazone, misonidazole, and metronidazole (Flagyl), with reduction potentials −282, −395 and −495 mV respectively. DNA damage, rate of chain elongation, and rate of replicon initiation were measured by hydroxyapatite chromatography of DNA. This method is based on the assumption that "breaks" in DNA serve as points of unwinding when the cells are subjected to alkaline solutions. Since a replication fork represents a "break" in DNA, unwinding will occur at this site. If cells (labelled overnight with $^{14}$C-TdR) are incubated for a short period with $^{3}$H-TdR, the isotope is incorporated at the growing fork, and subsequent incubation in "cold" medium allows the pulse to proceed from the single-stranded regions near the growing fork to double-stranded DNA further away. Thus it is possible to follow chain elongation during or after drug treatment by observing, over time after a pulse-label, the percentage of double-strand DNA containing $^{3}$H-TdR (Johanson & Rydberg, 1977). Also, the percentage of double-stranded DNA containing $^{14}$C-TdR is a measure of damage to parental DNA. The ratio of the total amount of DNA containing $^{3}$H-TdR to DNA containing $^{14}$C-TdR is a measure of DNA precursor incorporation after drug treatment.

The results reported here indicate that the effects of nitroheterocycles on chain elongation (in the absence of strand breaks) are distinct from those on initiation of new DNA replicons. Effects on incorporation generally precede other measurable effects and, unlike DNA strand breakage or cell survival, inhibition of incorporation is less influenced by ambient $O_2$ concentration. This in turn suggests different mechanisms of "toxicity" under aerobic and anaerobic conditions.

### MATERIALS AND METHODS

*Nitroheterocycles.*—Nitrofurazone (5-nitro-2-furaldehyde semicarbazone) was obtained from Norwich Pharmacal Company, Norwich, New York. AF-2 (2-(2-furyl)-3-(5-nitro-2-furyl)-acrylamide) was obtained from Dr G. T. Bryan, University of Wisconsin, Madison, Wisconsin. Misonidazole (R0-07-0582) was a gift from Dr C. Smithen, Roche Pharmaceutical Company, England. Metronidazole (Flagyl) was a gift from Searle Laboratories, San Juan, Puerto Rico. Drugs were prepared in medium containing 5% foetal calf serum before use from stock solutions (20–200 mg/ml) in DMSO (Sigma).

*Cells.*—Mouse L-cells were purchased from Grand Island Biological Company, Grand Island, New York. Cells were maintained in suspension culture in Joklic modified minimal essential medium with 10% foetal calf serum (GIBCO). Chinese hamster V-79 cells were grown as monolayers in Eagle's minimal essential medium supplemented with 15% foetal calf serum.

*Measurement of thymidine incorporation.*—The amount of TdR incorporated into DNA was measured as the ratio between the d/min of $^{14}$C-TdR (0·05 $\mu$Ci/ml, 80 Ci/mmol) incorporated after a 15min pulse before treatment (or a 20h incubation with 0·01 $\mu$Ci/ml $^{14}$C-TdR) and the d/min of $^{3}$H-TdR (2 $\mu$Ci/ml, 18 Ci/mmol) incorporated in a 15min pulse after drug treatment. This ratio was obtained from data using either radioactivity from whole cells precipitated on glass-fibre filters using cold 5% TCA, or total radioactivity present in the hydroxyapatite chromatography procedure. In the second method, the radioactivity in single and double-stranded DNA was combined to determine $^{3}$H-TdR and $^{14}$C-TdR incorporation respectively, and the ratio of the radioactivities then determined. Both methods gave quantitatively similar results

*Measurement of DNA single-strand breaks.*—Hydroxyapatite chromatography of DNA as previously reported (Olive & McCalla, 1977) was based on the technique of Ahnström & Erixon (1973). Briefly, cells labelled with $^{14}$C- or $^{3}$H-TdR were treated with nitroheterocycles, and resuspended in triplicate at $2 \times 10^4$ cells/ml. Cells were subjected to alkali lysis immediately in 0·02M NaOH and 0·98M NaCl for 60 min, neutralized by adding 1 ml of 0·02M $NaH_2PO_4$, and immediately sonicated for 15 s with a Branson Sonicator with a microtip. The sonicate containing 0·5% SDS was poured on to columns containing 150 mg of Biorad hydroxyapatite. The columns were washed with 3 ml of 0·012M Na phosphate buffer (equimolar mixture of $Na_2HPO_4$ and $NaH_2PO_4$) at pH 6·8. The single-stranded DNA was eluted with 3 ml of 0·12M Na phosphate buffer, and the double-stranded DNA with 1·5 ml or 0·4M Na phosphate buffer, pH 6·8. Whole samples were prepared for liquid scintillation counting by adding 10 ml Triton X-114 scintillation fluid

(Anderson & McClure, 1973) and 1·5 ml of water to the double-stranded sample. Recovery of radioactivity from the columns was >95%.

*Measurement of elongation.*—The procedure for labelling L cells with radioisotopes varied according to the experiment. When DNA strand breaks were measured in the same population used to determine chain elongation, L cells were labelled for 20 h with 5 nCi/ml $^{14}C$-TdR. It was then possible to follow elongation of DNA initiated in a 15min pulse of $^{3}H$-TdR given before or after drug treatment. A pulse given after drug treatment followed elongation into only those replicons that could still initiate DNA synthesis. However, a pulse given during treatment would be subject to DNA damage by the drug as well as effects on chain growth. In some experiments, rather than follow the complete time course of chain elongation, one time was chosen after the pulse for several drug concentrations. It is important to realize that the technique of hydroxyapatite chromatography is unable to distinguish between strand breaks in parental ($^{14}C$-labelled) or newly transcribed ($^{3}H$-labelled) DNA. A strand break in the $^{14}C$-labelled DNA will serve as a point of unwinding, and subsequent sonification will result in single-strand pieces which include the site of DNA damage as well as the opposite (perhaps intact) newly transcribed $^{3}H$-labelled DNA. Thus, whether chain elongation proceeds past a break (or alkali-labile damage) in the DNA cannot be determined using this technique.

To measure the rate of elongation in the absence of DNA breaks, a repair interval after drug treatment was followed by a 15min pulse of 2 $\mu$Ci/ml $^{3}H$-TdR and cells were incubated in nonradioactive medium containing 15% foetal calf serum for various periods (Johanson & Rydberg, 1977). L cells that were labelled overnight with $^{14}C$-TdR could then be examined simultaneously for DNA damage, incorporation and elongation (Fig. 1). Data were corrected for quenching by the external-standard ratio method and for "spillover" of isotopes by measuring the efficiency of counting of both isotopes in the $^{14}C$ and $^{3}H$ channels of a Beckman 8100 liquid scintillation counter. A computer programme was used to determine the actual disintegrations per minute in each channel.

*Measurement of cell proliferation.*—Exponentially growing mouse L-929 cells ($\sim 2 \times 10^5$ cells/ml) were incubated with nitroheterocycles either in suspension in medium equilibrated with $O_2$-free $N_2$, or in monolayers in a humidified $CO_2$ incubator. After treatment, cells were centrifuged (if in suspension) or removed from Petri dishes with trypsin and resuspended at a suitable density ($\sim$600 surviving cells/dish) for plating in duplicate. The surviving fraction was expressed as the ratio of the number of colonies to the number of cells plated, and the treated percentage of surviving cells was relative to the control fraction. In the situation where the same population of cells was used to determine the 4 endpoints, cells were labelled overnight with 5 nCi/ml $^{14}C$-TdR. There was no significant decrease in plating efficiency when L cells were incubated with both isotopes for this period.

*Measurement of colony size.*—As Chinese hamster V-79 colonies have better defined boundaries than L-929 cell colonies, they were used for measurement of colony size after treatment. Colony size was measured electronically by an Artek System Model 900 Cytotally. For each drug exposure, a 100mm Petri dish containing $\sim$600 V-79 cell colonies was analysed for the percentage of colonies of a particular size, a histogram was drawn, and the mean colony size per plate was determined.

FIG. 1.—Outline of the time course for the treatment of cultured cells with nitroheterocycles. H.C., hydroxyapatite chromatography; TdR, thymidine.

FIG. 2.—Effect of nitrofurazone on DNA damage, elongation, incorporation and cell proliferation. L-929 cells, labelled overnight with $^{14}$C-TdR, were incubated under aerobic conditions for 1 h with nitrofurazone dissolved in medium containing 5% FCS: –×–, cells analysed immediately after treatment. (a) DNA damage was measured using the technique of hydroxyapatite chromatography, by analysing the percentage of $^{14}$C-TdR in double-stranded DNA. The mean ±s.d. of triplicate determinations are indicated. (b) Elongation was measured by incubating L cells for 15 min with $^{3}$H-TdR, followed by 30 min in complete medium, drug treatment for 1 h, then analysis of the amount of double-stranded DNA present. Control level 40±4%. (c) Incorporation of $^{3}$H-TdR into both double- and single-stranded DNA treatment relative to the amount of $^{14}$C-TdR incorporated into double and single-stranded DNA before treatment. (d) The colony-formation assay was used to determine the percentage of cells surviving nitrofurazone treatment.

### RESULTS

In the experiment shown in Fig. 2, one population of mouse L-929 cells was incubated for 1 h with nitrofurazone under aerobic conditions. The various endpoints were assessed immediately and 90 min after treatment. DNA damage, measured as a decrease in the percentage of double-stranded DNA, was extensive for concentrations greater than 0·25mM (Fig. 2a). Treated cells examined for proliferative ability also showed a decrease in survival which paralleled strand breakage (Fig. 2d). Nitrofurazone treatment inhibited DNA synthesis, measured as a decrease in incorporation of $^{3}$H-TdR (Fig. 2c). The fate of elongating DNA was determined in this experiment by incorporating a small amount of $^{3}$H-TdR into exponentially growing cells. The pulse was allowed 30 min to move partially into double-stranded DNA before cells were treated with nitrofurazone. With concentrations of nitrofurazone greater than 0·25 mM, the percentage of double-stranded DNA (21%) fell below the value measured before drug treatment (40%; Fig. 2b). Since the pulse of $^{3}$H-TdR preceded drug

FIG. 3.—The rate of elongation measured using radiolabelled TdR incorporated before or after treatment of L cells for 30 min with nitrofurazone. (a) L cells were incubated with $^{14}$C-TdR for 15 min before treatment with –●– 0mM, –×– 0·2mM, or –△– 0·4mM nitrofurazone. They were then analysed for the percentage of double-stranded DNA at subsequent intervals. (b) L cells were incubated with –●– 0mM, –×– 0·2mM, or –△– 0·4mM nitrofurazone for 30 min followed by incubation for 15 min with $^{3}$H-TdR and analysis at subsequent intervals for the percentage of double-stranded DNA. The amount of incorporation relative to the control was determined using the ratio of $^{3}$H:$^{14}$C-TdR incorporated, and was 63% for 0·4mM and 88% for 0·2mM.

treatment, the effects on elongation may reflect 3 possibilities: (1) strand breaks are produced in the $^{3}$H-pulsed DNA (which apparently occurs only at high nitrofurazone concentrations), (2) the presence of breaks in parental DNA inhibits elongation, or (3) the presence of breaks in parental DNA does not inhibit elongation, but the hydroxyapatite technique does not distinguish between breaks in parental or newly transcribed DNA. Using this technique, it is not possible to distinguish between the second and third possibilities.

The problem of whether inhibition of elongation is the result of "template" damage or damage to the newly synthesized DNA was examined by the experiment in which a $^{3}$H- or $^{14}$C-TdR pulse preceded or followed nitrofurazone treatment (Fig. 3). In this experiment, L cells were labelled with $^{14}$C-TdR for 15 min. They were then incubated for 30 min with nitrofurazone followed by a pulse of $^{3}$H-thymidine. At various times after the second pulse, cells were analysed for the percentage of double-stranded DNA, using either the $^{14}$C or $^{3}$H radioactivity. If the effects on elongation were due only to strand breakage in the $^{14}$C-labelled DNA, the $^{14}$C data should reflect nitrofurazone damage, while the data for $^{3}$H should match the control data since the $^{3}$H-TdR was added *after* drug treatment. However, elongation of the $^{3}$H pulse after nitrofurazone treatment was also inhibited (Fig. 3) and the curves describing incorporation of both isotopes were similar, suggesting that the presence of strand breaks in the parental DNA was responsible (either directly or indirectly) for the decrease in the rate of chain elongation. The rate of chain growth appeared to be inhibited during treatment of L cells with nitrofurazone, but DNA damage was also accumulating during this period (Fig. 4a). When a pulse of $^{3}$H-TdR was given immediately after treatment of L cells for 1 h with 0·3 mM nitrofurazone, the rate of elongation was also inhibited, but to a lesser extent (Fig. 4b), corresponding to the repair of strand breaks in parental DNA. Two hours after drug treatment, there was no significant difference in the amount of double-stranded DNA in treated and untreated cells, although elongation was still depressed in treated cells (Fig. 4c). After 4 h, the amount of double-stranded DNA and the rate of chain elongation were similar in both populations (Fig. 4d).

FIG. 4.—Effects of nitrofurazone on DNA damage and elongation. Mouse L-929 cells were labelled overnight with $^{14}$C-TdR followed by treatment with (closed symbols) or without (open symbols) 250$\mu$M nitrofurazone. Elongation was followed by observing the processing of a pulse of $^{3}$H-TdR given immediately before (A), immediately after (B), 2 h after (C) or 4 h after (D) nitrofurazone incubation. The circles indicate DNA damage (using $^{14}$C-labelled DNA) and the triangles show the percentage of double-stranded DNA containing $^{3}$H-TdR (a measure of elongation).

FIG. 5.—Incorporation of $^{3}$H-TdR after nitrofurazone treatment. L cells were incubated for 4 h with nitrofurazone at the $\mu$M concentrations indicated. The ratio of the amount of $^{14}$C-TdR incorporated in a 15min pulse before treatment to the amount of $^{3}$H-TdR incorporated in a 15min pulse at various times after treatment was used as a measure of the DNA synthesis.

Inhibition of incorporation was found after incubation of L cells for 1 h with concentrations of nitrofurazone less than 0·125 mM, whilst there were no measurable effects on cell proliferation, chain elongation or DNA damage at this concentration (Fig. 2). DNA initiation was measured using the ratio of $^{14}$C- to $^{3}$H-TdR obtained by hydroxyapatite chromatography. Since the assay can be assumed to be equally sensitive for measurement of DNA damage and incorporation, it appears that initiation is more sensitive to nitrofurazone treatment than strand breakage. The inhibition of incorporation was unchanged after a 90min "repair" interval (Fig. 2c) and there was no significant increase in the relative rate of incorporation for up to 18 h after nitrofurazone (Fig. 5). Cell survival, being a late endpoint, was the same for cells assayed immediately after incubation with nitrofurazone, or 90 min later (Fig. 2d). However, these survival data do indicate that lysis of drug-sensitive cells has not occurred during the 90min repair interval.

A decrease was found in the mean colony size when Chinese hamster V-79 cells were incubated for 6 to 8 days in the presence of nitrofurazone, AF-2, metronidazole or misonidazole (Fig. 6). All histograms of colony size suggested a normal distribution after drug treatment. These data suggest that the mechanism of toxicity when the cells are incubated continuously with nitroheterocycles is growth inhibition by inhibition of initiation of DNA synthesis. The uniformity of colony size at any given drug concentration also suggests that, under chronic incubation, inhibition of DNA initiation occurs in all cells rather than in a sensitive population.

To examine the role of metabolites in inhibition of DNA synthesis by nitrofurazone, dense suspensions of L cells were

FIG. 6.—Decrease in colony size of cells grown for 6–8 days in the presence of nitroheterocycles. Relative cell survival did not fall below 0·87. $E_{1/2}$ is −265 mV for AF–2.

FIG. 7.—Effects of metabolites of nitrofurazone reduction on incorporation. $10^8$ L cells were used to reduce 20 ml of nitrofurazone under anaerobic conditions. The quantity of parent compound remaining at various times after the start of incubation was determined polarographically and the supernatant containing metabolites as well as parent compound was incubated with L cells. The inhibition of DNA synthesis was determined for nitrofurazone alone (–×–) and for the supernatant containing nitrofurazone and its metabolites (–○–). The total amount of nitrofurazone (parent compound and metabolites) was equivalent to the concentration before reduction of 0·8 mM.

used to reduce nitrofurazone under anaerobic conditions. The supernatant, after increasing periods of incubation, was analysed polarographically for the amount of compound retaining the intact nitro group. L cells were then incubated with this supernatant and the amount of inhibition of synthesis was determined (Fig. 7). The presence of metabolites of nitrofurazone apparently had no effect on the rate of DNA synthesis, as all the inhibition could be entirely accounted for by the amount of remaining parent compound.

Incubation with metronidazole for 1 h under aerobic conditions produced marked inhibition of DNA-precursor incorporation, but no significant effects on DNA damage, elongation or cell survival (Fig. 8). However, after incubation for 4 h under anaerobic conditions, all endpoints indicated damage to L cells. With misonidazole, a 1h aerobic incubation had a small effect on initiation, whilst 1 or 4 h under anaerobic conditions showed increasing DNA damage for all endpoints (Fig. 9). For both metronidazole and misonidazole, the rate of incorporation of $^3$H-TdR did not increase after drug treatment for at least 4 h (data not shown).

FIG. 8.—Effect of metronidazole on DNA damage, elongation, incorporation and proliferation. L cells, labelled overnight with $^{14}$C-TdR, were incubated for 1 h with metronidazole under aerobic conditions (–×–), or 1 (–○–) or 4 h (–●–) under anaerobic conditions. Elongation was measured by incubating L cells after metronidazole treatment in $^{3}$H-TdR for 15 min, followed by incubation in drug-free medium for 60 min, then analysis of the percentage of double-stranded DNA. See caption to Fig. 2 for further details.

### DISCUSSION

The technique of hydroxyapatite chromatography has been applied in a new way to the analysis of effects of nitroheterocycles on several aspects of DNA damage. Damage to parental DNA can be determined, as well as the rate of initiation and elongation of new DNA. Not only can the same population of cells be used for all assays, but data using one technique can be manipulated to give information on 3 of the endpoints, thus avoiding the problem of comparing assays of different sensitivities.

With the 3 nitroheterocycles, drug concentrations or times of incubation were found which inhibited DNA-precursor incorporation, with little or no damage to DNA or proliferative ability (when incubation takes place under aerobic conditions). With nitrofurazone, cell survival using the colony-formation assay generally paralleled the decrease in DNA initiation. With metronidazole and misonidazole, initiation decreased to a greater extent than reflected by survival data, when cells were exposed in medium equilibrated with air.

Inhibition of DNA synthesis by nitroheterocycles apparently involves effects on both the processes of initiation (precursor incorporation) and chain elongation. Since inhibition of chain elongation can result from the presence of strand breaks in parental DNA, the technique of hydroxyapatite chromatography can give information on elongation only in the absence of strand breaks. Under these

FIG. 9.—Experiments as for Fig. 8, but using misonidazole in place of metronidazole.

conditions, either the rate of elongation is unaffected (at low drug concentrations) or it is slightly depressed (after a short "repair" interval (see Fig. 4c)). However, effects on initiation generally occurred at lower concentrations and were more slowly repaired than effects on DNA damage and elongation. Indeed, inhibition of initiation would account for the decrease in relative cell survival seen when cells were incubated for 8 days with nitroheterocycles (Adams *et al.*, 1976). The decrease in mean colony diameter was not accompanied by a decrease in cell "survival" until the colony size became so small that the cells were considered to have lost proliferative ability (Fig. 6). A correlation also exists between the electron-affinity of the nitroheterocycles shown in Fig. 6 and the concentration required to decrease the colony size to 50% of the control size.

Although elongation proceeds at a rate similar to the control when breaks are rejoined, this necessitates repair only of DNA damage causing strand breakage. In fact, pyrimidine dimers due to UV damage persist within human cell DNA, and their presence does not inhibit synthesis of normally sized DNA (Buhl *et al.*, 1973). Thus, although strand breaks may be rejoined and chain elongation proceeds, the damage inhibiting DNA incorporation remains apparently unrepaired for long after treatment. This damage may ultimately cause loss of proliferative ability.

The process of initiation (rather than elongation) has also been shown to be more sensitive to ionizing radiation, using hydroxyapatite chromatography (Johanson & Rydberg, 1977) gradient centrifugation (Painter & Young, 1975; Makino & Okada, 1975) and autoradiography

(Watanabe, 1971). On the other hand, the alkylating agent N-methyl-N-nitrosourea prevented an increase in the mol. wt of newly synthesized DNA (Roberts, 1975). Painter (1978) recently reported that 4-nitro-quinoline-N-oxide inhibited DNA initiation by 50–60% at a concentration which did not produce effects on DNA elongation or stranded breakage. It is interesting to note that incorporation did not decrease with time after treatment of L-929 cells with nitrofurazone, as was observed for other classes of DNA-damaging agents (Painter, 1977).

While inhibition of initiation may account for the toxicity found when L cells are incubated in a chronic fashion with nitroimidazoles, much greater toxicity (DNA damage and cell killing) was seen when cells were incubated under anaerobic conditions, and this toxicity was not reflected in greater inhibition of DNA synthesis. Under anaerobic conditions, intermediates of nitro-reduction probably play a more important role in cell inactivation than inhibition of DNA synthesis. The previous paper indicated that an intact nitro group was required for inhibition of DNA synthesis. Since metabolism of nitroheterocycles is greatly increased under anaerobic conditions, it appears likely that the parent compound or nitro anion radical is responsible for the effects observed here on DNA synthesis. Also, products of metabolism of nitrofurans by L cells were ineffective in inhibiting DNA synthesis.

Previous results (Olive, 1976) indicated that nitrofurazone inhibits ATP synthesis in L cells incubated for 2 h at concentrations over 100 $\mu$M. This inhibition occurred under both aerobic and anaerobic incubation, similar to the effects on inhibition of DNA initiation, suggesting that decreases in the level of ATP inhibit DNA synthesis. The relevance of effects of ATP and glucose utilization in *E. coli* to inhibition of nucleic acid synthesis was recently examined by Lu & McCalla (1978). Using 4 nitrofurans, no obvious correlation was found between the energy state of the cell and inhibition of DNA or RNA synthesis. Also, results using AF-2 (furylfuramide) suggested that the inhibitory effects of this drug were due to metabolites only, whilst for nitrofurazone inhibition of macromolecule synthesis occurred both in *E. coli* B/r and a mutant of this strain which was deficient in the ability to activate nitrofurans. However, results shown in Fig. 6 suggest that, in mammalian cells, AF-2 can inhibit DNA synthesis under aerobic conditions in a manner similar to nitrofurazone.

Nitroheterocycles, including metronidazole and misonidazole, are currently in use clinically as hypoxic cell radiosensitizers. At the plasma levels one expects to achieve in patients (0·25mM misonidazole and 1·2mM metronidazole) little inhibition of DNA synthesis, and consequently little drug cytotoxicity to aerobic cells, would be predicted, as no growth inhibition was seen in Chinese hamster V-79 cells or mouse L cells incubated for 8 days with these concentrations under aerobic conditions. The increased toxicity under hypoxic conditions does, however, suggest the possibilities of preferential antitumour effects.

The author is indebted to Barb L. Thomas for excellent technical assistance and to Dr Ralph Durand for helpful discussions. This investigation was supported by Grants Number CA 24519 and CA 06973 awarded by the National Cancer Institute, DHEW.

REFERENCES

ADAMS, G. E., CLARKE, E. D., JACOBS, R. S. & 4 others (1976) Mammalian cell toxicity of nitro compounds: dependence upon reduction potential. *Biochem. Biophys. Res. Commun.*, **72**, 924

AHNSTRÖM, G. & ERIXON, E. (1973) Radiation induced strand breakage in DNA from mammalian cells. Strand separation in alkaline solution. *Int. J. Radiat. Biol.*, **23**, 285.

ANDERSON, L. & MCCLURE, W. (1973) An improved scintillation cocktail of high solubilizing power. *Anal. Biochem.*, **51**, 173.

BUHL, S. N., SETLOW, R. B. & REAGAN, J. D. (1973) Recovery of the ability to synthesize DNA in segments of normal size at long times after ultraviolet irradiation of human cells. *Biophys. J.*, **13**, 1965.

FUSKA, J., FUSKOVA, A. & JURASEK, A. (1974) Effect of 5-nitrofuran derivatives on the uptake of labelled nucleic acids and protein precursors in the acid-insoluble fraction of Ehrlich ascites tumor cells. *Neoplasma*, **20**, 171.

JOHANSON, K. J. & RYDBERG, B. (1977) The effect of $^{60}$Co gamma-radiation and hydroxyurea on the *in vivo* chain growth of DNA in crypt cells of the small intestine of the mouse. *Int. J. Radiat. Biol.*, **31**, 441.

KIKUI, M. (1968) Antitumor activity of nitrofuran derivatives against Ehrlich ascites tumor. *Med. J. Osaka*, **19**, 127.

LU, C. & MCCALLA, D. R. (1978) Action of some nitrofuran derivatives on glucose metabolism, ATP levels, and macromolecule synthesis in *Escherichia coli. Can. J. Microbiol.*, **24**, 650.

MAKINO, E. & OKADA, S. (1975) Effects of ionizing radiations on DNA replication in cultured mammalian cells. *Radiat. Res.*, **62**, 37.

NAKAMURA, S. & SHIMIZU, M. (1973) Inhibition of synthesis of macromolecules in *Escherichia coli* by nitrofuran derivatives. I. (5-Nitro-2-furyl) vinylpyridines. *Chem. Pharm. Bull.*, **21**, 130.

OLIVE, P. L. (1976) *Damage of Mammalian Cell DNA by Nitrofurans*. Ph.D. Thesis, McMaster University, Ontario.

OLIVE, P. L. (1979) Inhibition of DNA synthesis by nitroheterocycles. I. Correlation with half-wave reduction potential. *Br. J. Cancer*, **40**, 89.

OLIVE, P. L. & MCCALLA, D. R. (1977) Cytotoxicity and DNA damage to mammalian cells by nitrofurans. *Chem. Biol. Interact.*, **16**, 223.

PAINTER, R. B. (1978) Action of 4NQO adriamycin and ethylenemine on DNA replication in HeLa cells. *J. Supramol. Struct.*, **2**, 96.

PAINTER, R. B. (1977) Rapid test to detect agents that damage human DNA. *Nature*, **265**, 650.

PAINTER, R. B. & YOUNG, B. R. (1975) X-ray induced inhibitions of DNA synthesis in Chinese hamster ovary, human HeLa and mouse L cells. *Radiat. Res.*, **64**, 648.

ROBERTS, J. J. (1975) Repair of alkylated DNA in mammalian cells. In *Molecular Mechanisms for Repair of DNA*. Ed. P. C. Hanawalt & R. B. Setlow. New York: Plenum Press. p. 611.

WATANABE, I. (1971) Radiation effects of DNA chain growth in mammalian cells. *Radiat. Res.*, **58**, 511.